# The Hospital Nursing Supplement

**Published:** 1895-12-14

**Authors:** 


### "The Hospital" Nursing Mirror.

Being the Extra Nursing Supplement of "The Hospital" Newspaper.

[Contributions for this Supplement should be addressed to the Editor, The Hospital, 428, Strand, London, W.C., and should have the word "Nursing" plainly written in left-hand top corner of the envelope.]

### News from the Nursing World.

### OUR NEEDLEWORK COMPETITION.

Parcels for competition and subsequent distribution to sick people in hospitals should reach the office on December 16th and 17th. Each parcel should be addressed "Needlework Competition," care of the Editor of The Hospital, 428, Strand, London, and should contain the name and permanent address of the sender.

### NURSES AT WESTMINSTER HOSPITAL.

Amongst other privileges enjoyed by the nursing staff of Westminster Hospital ranks the use of a charming convalescent home. It is very pleasantly situated at Eastbourne, and thither go nurses and probationers requiring days or weeks of rest. Two or three days change often fits a tired nurse for resuming her work, whilst a month may be required by one who has had an injury or illness. The home is free to the whole of the private and hospital nursing staff, and it does much to keep the average of health good. The management at Eastbourne aims at making the home home-like, and it has succeeded. When the rooms are not all needed by the Westminster nurses, it is possible for nurses from other hospitals to gain admittance provided they are thoroughly well recommended. These nurses pay a moderate weekly inclusive fee. Like other things at Westminster, this convalescent home was established without any preliminary publicity, and few people know the amount of practical good which it has conferred on the nurses.

### MISS NIGHTINGALE'S OPINION OF REGISTRATION.

The idea of State registration for nurses apparently does not commend itself to Miss Nightingale, for she has recently expressed herself as altogether averse to any system of public registration, which she considers could not fail to prove misleading to the public itself. Miss Nightingale fails to see any similarity between the registration of medical men and that of nurses. People do not choose a doctor only because his name is on the medical register, but, on the contrary, she thinks that many people understand so little of nurses that they would employ a woman simply because her name appeared in a public register.

### NURSING SISTERS FOR ASHANTEE.

The "Coromandel" steamed out of the dock gates on Saturday amidst ringing cheers from crowds of spectators, and inspiriting music of the military bands. Besides the troops the "Coromandel" carried a small party of Her Majesty's Nursing Sisters who have been selected for active service under Miss Gray. This lady, recently superintendent of the hospital of the Coldstream Guards, has had valuable experience in military nursing, and did good work in the Zulu and Egyptian campaigns. Miss Gray received the Royal Red Cross for heroic service in Egypt, where she won universal esteem and regard, and she was also decorated with the Zulu war medal and the Egyptian war medal and cross.

### UNIVERSAL UNIFORM.

The Englishwoman who writes to us this week from the north is to be congratulated on her excellent letter, which is given elsewhere. Her vigorous common sense is quite refreshing, and appeals to many nurse-readers who are heartily tired of the attitude taken in various quarters with regard to nurses' dress. It seems to be considered creditable for nurses to be helpless in this matter, whereas they are certainly free either to discard outdoor uniform altogether, or, better still, to wear it so neatly, becomingly, and modestly that they will be in appearance as well as character true nurses. Suggestions for a general uniform for all sorts and conditions of nurses will receive but little favour from the best type of women.

### A PLEA FOR OLD CUSTOMS.

Some of our readers seem inclined to confuse the opinions of our correspondents with those held by ourselves, in spite of the head-note to the column of "Everybody's Opinion." We have, for instance, been assailed this week with the reiterated inquiry, "Surely you don't disapprove altogether of Christmas decorations in hospital?" Most assuredly we do not desire to condemn them, for we have personal knowledge of the great pleasure which they give to the patients and to their friends. To those who have so few enjoyments, and whose homes are entirely devoid of beauty, Christmas decorations have a charm little understood by the inhabitants of luxurious houses. We have certainly called attention on many occasions to the folly of decorations being permitted to absorb time, money, and strength, which can be ill-spared by nurses. Our object in doing so is not to check the kind, unselfish efforts of residents in hospital, who surely deserve every encouragement, but rather to obtain for them liberal support from those who live outside hospitals. So long as decorations and Christmas festivities are kept within reasonable bounds they are productive of good, and not evil. That elaborate adornments harbour dust cannot be gainsaid, but if they are only left up for a few days the quantity accumulated is imperceptible. The remarks made by an esteemed correspondent last week on "decaying vegetable matter" hanging day and night on ward walls can hardly be seriously intended. We doubt the possibility of such uncleanliness prevailing in any decent institution. Walls tiled with pictures as at Paddington Green Children's Hospital, or artistically coloured as they are in many others, leave little room for extra embellishment; but colour-washed walls, such as those at Poplar Hospital, might be made beautiful for one short week at Christmas, more consistently than being, as at present, permanently adorned by hanging pictures, and we hope that if the energetic chairman of the Poplar Hospital is assured that the decorations shall not be permitted to decay, he will think more kindly of them.

### THE NURSE AS CLINICAL CLERK.

The benefit of trained nursing is appreciated not only by the patient, but by the doctor, and probably by him in no single point more than in this—that from the trained nurse he receives a reliable and intelligible account of the progress of the case during the interval between his visits. Few people would believe till they have experienced it how difficult it is to obtain from friends and relatives anything like a connected story of the day's proceedings. Each attendant has her own tale to tell, and all these little stories have to be dovetailed together by the doctor, who often finds it no easy task. One person goes by the church clock, another by the clock on the chimney-piece, another by her own watch, which she knows is just five minutes late, for she set it by Big Ben only a week ago, and it always loses five minutes a week! All these exasperating details are apt to be poured out into the poor doctor's ears in answer to a simple question as to the time a particular pudding was given. Endless notes and memoranda written on different sized scraps of paper have to be compared, and it may appear as if the unfortunate pudding had vanished into space, till at last a housemaid is bound to confess that she took it in "just before Miss Mary came in," and to tell with pride and satisfaction that "dear lamb, she sat up and took it quite greedy like." Sat up, indeed! A nice look out for a case of pneumonia! And this just after a row of sisters had been assuring the anxious doctor that they had been "so careful." Now with a trained nurse all this puzzle is avoided. Whatever else a trained nurse may do, she can at least give a reasonable description of what has been going on, and make definite statements as to times and quantities. This is part of her training, and in the picture published in the Special Supplement we see her at her daily task of "taking notes," all of which records of her cases will be carefully criticised by the training sister later on.

### WORKHOUSE INFIRMARY NURSING ASSOCIATION.

With regard to the infirmary of St. George's-in-the-East, the committee of the Workhouse Infirmary Nursing Association, after much consideration, were forced to place before the Guardians the fact of their inability to supply them with more nurses, in view of the increasing appeals from country unions for assistance in their efforts to inaugurate a system of trained nursing in their infirmaries. For nearly ten years nurses have been sent, at considerable expense, by the Workhouse Infirmary Nursing Association to St. George's-in-the-East. Altogether, about a hundred have been supplied, besides several to the sick wards in the workhouse, of these no less than seventy-five were trained at the expense of the association. The twenty-six nurses who are at St. George's-in-the-East at present have not been withdrawn, but the association is obliged to discontinue to supply candidates to any further vacancies.

### BADGES AT SALISBURY.

The committee have introduced badges for the nurses at Salisbury Infirmary. Those of the matron and night superintendent are in blue enamel and silver. The head nurses, whose position corresponds with that of ward sisters, have silver badges, and the probationers have bronze ones. On each appear the arms of the city of New Sarum, pierced with a band bearing the motto of the hospital, "The sick and needy shall not alway be forgotten." This device hangs from a bar on which is engraved, "Salisbury Infirmary." The committee have given many proofs of their interest in the welfare of their nursing staff, and appear to have every wish to forward the matron's wishes in the matter of training.

### NIGHT AND DAY DUTY.

A paragraph with this heading in a recent issue of The Hospital has brought us some interesting correspondence, and an experienced superintendent has written her own personal experience of this style of nursing. After thanking us for what she kindly calls our "extremely pertinent remarks," she says that she has long wished to enter a protest against the iniquitous custom of using the same nurse for day and night duty, which she has seen result in literally wearing out a willing nurse. Speaking of a surgeon whose cases of hysterectomy and ovariotomy are always put under the care of a single nurse, she explains that for three days and nights scarcely any sleep has been possible, and even after that only broken rest in the patient's room is attainable. Another correspondent tells of a surgeon who characterised as absurd her own objection to having in her Home a patient who was to be tended by one nurse, who would be on duty for a period varying from forty-eight to seventy-two hours, without any prospect of sleep and very little of rest during that period. We cordially agree with our correspondent's vigorous remarks, and trust that the doctors and matrons who still permit such inhuman treatment of nurses, and such obvious injustice to patients, will soon see things from this superintendent's point of view.

### PRINCE ALFRED HOSPITAL, SYDNEY.

A very high degree of excellence has been attained in the nursing department of this hospital, which now possesses a model training school. The class of candidates who apply to be received as probationers is also eminently satisfactory, and the educational advantages offered appear to be highly valued. Miss McGahey (the matron) has succeeded in making the nursing department worthy of the fine hospital to which it is attached.

### SHORT ITEMS.

The fourth annual meeting of the Milngavie Nursing Association was recently held in the Burgh Hall.—It is proposed by the Blackburn Guardians to appoint two trained nurses to visit the sick poor in their homes.—A committee has been appointed to confer on the possibility of securing a trained district nurse for Puckeridge.—Nurse Roberts has been engaged by the Levenshulme District Nursing Association to begin work there at once.—Nurse Prior, formerly sister of the Wesleyan Mission at Saltash, was presented by the members of the Mothers' Meeting with a handsome copy of Wesley's Hymns; Nurse Prior is now working at Plymouth.—Steps are being taken by the Guardians to reorganise the nursing department at Clapham Workhouse Infirmary.—A house on Stepney Green has been taken and furnished by Lady Algernon Gordon Lennox for a nurse, who will devote her time to the sick poor in the neighbourhood. The first lady appointed to this post is Miss Thompson, for many years Sister Charlotte at the London Hospital.

### Elementary Physiology for Nurses.

By C. F. MARSHALL, M.D., F.R.C.S., late Surgical Registrar Hospital for Sick Children, Great Ormond Street.

### XIX.—THE EYE (*Concluded*).

### APPRECIATION OF COLOUR.

IN sound we distinguish between waves which are more or less rapid; the longer more slowly vibrating, causing a deep, bass note; the shorter and more rapid waves causing higher or treble notes. So it is with light, we can distinguish waves of different length, and these differences are appreciable to ourselves as colour.

By means of a prism we can split up rays of light into their component colours, forming a spectrum. These colours are in definite order, red being at one end of the spectrum and violet at the other; yellow, green, and blue being intermediate. Each colour is of a different wave length to the others, red having the longest waves and violet the shortest. White light is thus made up of rays of all colours, and the mixture gives rise to the effect of white. The colours of the spectrum are well seen in a rainbow.

*Complementary Colours.*—After looking for a time at an object the eye is fatigued. If we look at a white spot on a black ground for a time, and then suddenly look at a moderately lighted surface, say the ceiling of a room, we shall see a dark spot. And if a coloured surface is looked at the complementary colour will be seen. The complementary colour is white minus the particular colour concerned, *i e.*, the colour which will make white with the colour in question. For instance, red and green are complementary colours, for red and green light when brought together by a prism will make white light. So orange and blue are complementary. Thus when a red spot is looked at for some time, and the eye then looks at the ceiling, a green spot will be seen there. The explanation being that the eye becomes fatigued for red, and when rested sees its complementary colour.

*Colour-blindness.*—In some people the range of colour perception is less than normal, and such people are called colour-blind. The most usual form is inability to distinguish between red and green. The cause is very uncertain, and is too complicated to be discussed in these lectures.

The retina consists of several layers. The innermost is the layer of nerve fibres from the optic nerve. The outermost layer is that of the rods and cones. Intervening between these are several layers called nuclear and molecular. The most important layer is that of the rods and cones, and this layer is probably connected indirectly with the fibres of the optic nerve, although such a connection is difficult to demonstrate. In the retina are certain parts of special interest. The blind-spot is the entrance of the optic nerve, and is insensitive to light; hence the nerve fibres themselves, curious though it may seem, are blind. The yellow spot is that part of the retina in the axis of direct vision, or the optic axis. In this the cones are very abundant, but there are no rods, and the other elements of the retina are absent, except the nerve fibres. The yellow spot is the seat of most acute vision, for it falls in the direct line in which we look at an object. Therefore, the cones are the most sensitive part of the retina, for it is these which are most abundant in the yellow spot.

It is interesting to note that light must traverse the thickness of the retina to reach the rods and cones, because these are the outermost layer. The blood-vessels of the retina enter with the optic nerve, and are distributed internally to the layer of rods and cones. Hence shadows of the blood-vessels must be formed by the light passing through them to the rods and cones. These can be actually seen in ourselves by entering a dark room with a candle held below the level of the eye, and moved about while the eye gazes at a blank wall. The vessels will be seen as a radiating network on the wall. They are known as Purkinje's figures.

The effect of pressure on the optic nerve is to cause a sensation of light, for the optic nerve is accustomed to carry messages of this kind only; consequently any stimulation of it or blow on it causes a sensation of light. Hence the seeing of "stars" when a blow on the eye is received.

### VISION WITH TWO EYES.

Although there is a separate image formed by each eye, yet the brain in its normal condition is conscious of only a single image. This is again the effect of experience. The outer side of one eye must work with the inner side of the other eye, and if the light is so directed that light from one object falls on points of the retina not accustomed to work together we shall see double. But by experience each half of each eye works together with the opposite half of the other eye, and the brain by experience learns to associate the points of the retina which receive the image of one eye with the points of the retina of the opposite half of the other eye, thus combining the two images. If we hold one finger at arm's length, and another closer to the face, and focus our eyes for either finger, the other will appear double. The explanation is that, when the eyes are focussed on one finger, light from the other finger will fall on two non-corresponding parts of the retinas of the two eyes, *i.e.*, on parts which are not accustomed to work together. The images in the two eyes are not identical, as we can see by looking at a solid object a little distance off, and closing each eye alternately; a different view is obtained by each eye. By this means the effect of solidity is obtained by the brain combining the two views obtained from both eyes. Distance is estimated by the degree of convergence of the two eyes when focussed on an object. When a man loses one eye this power of estimating distance is lost to a great extent, but is regained largely by the muscular sense of the ciliary muscle during accommodation.

### Probationers at York County Hospital.

THE prizes and certificates gained by the nurses and probationers of the York County Hospital were distributed by the Very Rev. the Dean of York, chairman of the House Committee. Nurse Emily Tute, who had full marks both in anatomy and nursing, carried off the first prizes; Nurse Annie Mountain took first prize for physiology, and also a prize for the highest aggregate number of marks. Probationers Bertha Relton, nursing prize (full marks); Phillipa Keith, second anatomy; Annie Morgan, second anatomy; Margaret Hindmarsh, second physiology prizes. The lecturer on anatomy was Mr. S. Shann, L.R.C.P.Lond., M.R.C.S.; on physiology, Mr. C. Hodgson, M.R.C.S., L.R.C.P.Lond.; on nursing, Mr. R. H. Luce, M.B., B.C., &c. The Dean, in speaking to the nurses, remarked on the very high percentage of marks obtained.

### County Nursing Association.

AT a recent meeting at 4, Bedford Square, a discussion took place as to the advantage of some common principle for associations which organise rural nursing. The Earl of Winchilsea, who was in the chair, spoke of the reasons which made it desirable to have some general standard of training and professional inspection.

### What Christmas Means to Hospital Officials.

General Anticipations.

"Christmas will soon come" is uttered joyously by childish voices, even by those poor little folks who have hitherto but small experience of pleasant things. Perhaps in them also arises a hope that the festival which is only a name may prove in some way advantageous. To poor householders the thoughts of Christmas too often brings only foreshadowing of suffering and distress, whilst more prosperous people associate with the season many prospects of enjoyment, leavened perchance by such drawbacks as large expenditure, children's protracted holidays, frozen water-pipes, and illness, or, at the least, indisposition, in the family circle. "Everyone is so busy at Christmas" is a common saying in private households. What else can it then be in hospitals?

Aspect in Hospital.

It is generally the festival itself which is held responsible for making everybody outside a hospital "so busy" at Christmas; but inside the wards the workers are already sufficiently occupied beforehand. At this season there is often a great increase in the number of patients, and the serious character of the medical cases admitted, places an extra strain on the resources of the institution. Probably some members of the staff may also be on the sick list, which reduces the available workers. Hence Christmas makes hospital people more than busy, for they are already busy independently of the festival, and the seasonable preparations for it form a serious addition to their other duties.

Committees and Governors.

Those connected with hospitals as governors or members of committee frequently ignore the responsibilities attached to their office, but a steadily-increasing number are at last beginning to realise that they ought to take a practical interest in much that goes on within the hospital walls besides the mere treatment of the sick. To walk through the wards on Christmas Eve accompanied by personal friends, and talk of what is done in "my" hospital is not the whole duty of any one of these gentlemen, even if he also personally conducts another party on Christmas Day to witness the serving up of the dinners to the patients. This commendable supervision needs to be preceded by practical help. Timely gifts in coin or in kind might enable the donors to taste the true luxury of charity while lightening the annual drain on the pockets of sisters and nurses, which is a serious matter to many who are not well off.

Male Officials.

The male officials, from the secretary down to the youngest porter, do a considerable share of the extra work which Christmas brings in its train, and in that way ease the burden which would otherwise fall on the nursing staff, and they do it for the most part very willingly. Beyond a Christmas dinner, officially recognised, and a few unacknowledged "tips," the porters have little to compensate them for the extra duties entailed by the clearing up of piles of evergreens, and the receipt and delivery of parcels, &c. Yet it is most unusual to hear any grumbling from them, and probably without the staff thinking much about it, the true spirit of Christmas is abroad in institutions for the sick, and in the presence of helplessness and suffering all do their utmost.

Christmas for the Patients.

"I suppose you are beginning to think about Christmas?" is asked by visitors a week or two before the day itself, as they look round and see no immediate sign of preparations. Probably the charge nurse replies to the effect that she began to *think* of Christmas as soon as she returned from her annual summer holiday, but she does not add that she has also been saving up for it. Experienced workers do not talk much of their plans beforehand nor allow any signs of decoration to intrude into the midst of the routine work till a week or ten days before Christmas; then preparations are commenced, and all odd moments are devoted to planning out novel adornments. Perhaps only nurses themselves can rightly estimate the amount of pleasure which they secure to patients who spend a Christmas in hospital. Of course, some are too ill to take any interest in aught beside their own condition; and these cases form the sad side of the scene, but they are seldom disturbed by their neighbours, for each is sufficient to himself. With the attention of a special nurse and the presence of his own friends, a "bad case" need take no harm from the festive preparations around him. The majority of patients enjoy unfeignedly all that is done to make the Christmas spent in hospital one of the most memorable events of their lives. Their keen interest in scraps of gossip about what is going on in other wards makes them desirous their own should be the prettiest.

The Adult Patients.

Amongst the men there are always some who are clever with their hands, such as sailors, mechanics, or old soldiers, who plead for "something to do." The busy nurse is glad to hand over long strips of wreathing to them, and sometimes finds her pupils more skilled than herself. They, having nothing else to do, take infinite pains and time over their work, and point out pridefully to wife or mother the exact amount done by themselves. "It helps to pass the time," explains a robust man, disabled temporarily by an accident, and his cheery industry proves contagious, until nurse is driven to requisition the services of some juvenile convalescent to keep her assistants supplied with materials for their work.

Even the poor medical cases take an interest in the appearance of their ward, and volunteer, "I dessay I can do a bit for you, nurse, after the doctor's been round." The patients do not, however, work only for the honour of the ward, or "to please nurse," but they have a thought for the visitors' day, when their own friends and relations will be admitted to praise and admire, which they generally do very heartily.

The women also help on the preparations, though they are seldom as keen on this work as the men, but equally pleased with results. Possibly the daily round of their ordinary lives is made up of such incessant toil that they are content to lie idle, and care less for the temporary distractions than the men.

The Children.

However cordial the interest which adults take in Christmas preparations, it cannot equal that which is exhibited by the juvenile patients. To the latter Christmas in hospital is indeed a glimpse of Paradise—a period into which is concentrated pleasures never before within their reach. Everyone bestows kindly notice on them, and strives to give them pleasure; and besides this, the youngsters make many diversions for themselves out of the actual preparations. Every parcel that comes into the ward has a possibility of hidden treasures, and many are the wonderful things whispered into mother's ear as to what "we're a goin' to 'ave at Christmas."

From grave visiting doctor to first-year student everyone finds time to "hope the children are enjoying themselves," and to do what they can to secure this end. If anyone remarks to the nurses, "How tired you must be when it's all over," they generally reply bravely, "But, indeed, it is well worth the doing."

Extraneous Assistance.

"Why don't they get more help from outside?" Well, there are many people who could devote one or two days to assisting in matters which nurses could gladly delegate to them. There are parcels to be packed (for patients like to receive their gifts duly enclosed and directed), decorations to procure and place, Christmas presents to make, beg, or buy, for nowadays every sister or charge nurse likes to have a token of some sort for each of her patients. But abnormally busy as they are themselves, the nurses know only too well how few people are really helpful, and those who need many instructions and explanations, or are too prone to suggestiveness, hinder rather than help.

THE MATRON.

The doctors, the committee, and the secretary each get credit for their kindly interest and their exertions to give the hospital staff a happy Christmas, but few people realise how largely their enjoyment of the festival depends on the matron. Her share in the work cannot be estimated, because she alone knows its extent. Besides providing for the material comforts of her nursing and domestic staff she has to see that their services are duly distributed, and that the care of the sick is not relaxed. The matron is expected to bear in mind details which everyone else seems excused from remembering, and she has to supplement supplies of all kinds at the shortest notice. She has to see that gifts are suitably acknowledged and strangers properly welcomed, and to be practically always on duty during the Christmas season. For her reward she gets merely a careless, "Think everything has gone off well," from one of the responsible officials. The nurses are sure of gratitude from their several patients, and the matron adds her praises to sisters and nurses for all that has been done by them; she alone getting most precarious appreciation, and often a vast deal of criticism.

PRACTICAL ASSISTANCE.

The generosity shown to the patients by the medical and nursing staff has become proverbial in many hospitals, and it is often altogether out of proportion to their modest means. Surely it is time that more help came from others—from those who cannot give personal service to the sick and the poor?

How few people trouble to add, "Who provides it all?" to their complimentary remarks on tastefully-draped colours or daintily-spread Christmas tea-tables.

Few hospitals have funds to spare for such luxuries, and, even if they had, it is difficult to say how far public money should be used to buy crackers and holly. Individual liberality would meet the matter, and special donations for Christmas expenses would be an appropriate thank offering for present blessings from those to whom "A Happy Christmas" is no empty phrase.

### Where to Go.

ST. MARY'S BOLTONS SCHOOLS, GILSTON ROAD, S.W.—The jumble sale organised by Mrs. Wood and Miss Paget for the benefit of the funds of the Trained Nurses' Club will take place in these schoolrooms on December 20th, at half-past three. Contributions of any kind will be gladly received, or they can be collected by a cart on Thursday, 19th inst., if a postcard is sent beforehand to Miss Stubbs, St. Mary's Schools, Gilston Road.

LONDON HOSPITAL CHILDREN'S CHRISTMAS TREES.—Monday, December 30th, at two p.m.

### Presentation.

ON leaving the South Devon Hospital to take up her new duties at the Plymouth Royal Eye Infirmary, Miss Edith Olphert was presented by the nursing staff with a clock, ebony brush and hand glass with silver monogram, and by her patients with six silver teaspoons. She takes many good wishes with her to Plymouth.

### Miss Nightingale in Buckinghamshire.

CONTRIBUTED.

WE were strolling about in the lovely lanes near Claydon last year, when someone announced that Miss Nightingale was coming down on a visit, and we turned our steps towards the Hall with a lingering hope that we might get a glimpse of our heroine. We were only just in time to see a quiet brougham drive up to the main entrance, and from it alighted a lady and two maids, and we knew without asking that the "lady with the lamp," Florence Nightingale was here. Then we retraced our steps satisfied, and met, coming along the avenue, an elderly man escorted by a sprightly girl of twelve, with soft brown eyes and shining hair. He was carrying a bunch of beautiful flowers, and had a pink and green buttonhole. "Good evening," we said. "How goes the day?" "Why, 'tis past an' over," replied the hero, glancing at the crimson west, "but I shall remember it as long as I live." "Is it one of the red-letter days? You are looking as smart as if you were going on parade." "No more parades for me," he answered sadly. "Look at this," holding up for our inspection the charming bouquet. "Now don't you call that a pretty nosegay? I bin' a lookin' arter my indoor plants a bit extra, for a month or so, thinkin' to pick the best blooms when Miss Nightingale come down, but I had to beg the rare ferns," and his face suddenly lighted up. "Will Miss Nightingale accept your flowers?" "I hope so," said he, gravely. "She's a rare kind lady; allers good to soldiers. I suppose you know, ma'am, that I've seen service wi' the colours?" "Have you been abroad?" "Yes; eleven years in Gibraltar and India. After the Mutiny I was invalided home an' discharged, an' only for Miss Nightingale I should not be steppin' out like this. She asked one o' the best doctors in London to come down an' see me, and he helped me wonderful. I shall never be quite cured, but then some on us must fight for the Queen. An' I ha' got my cottage and my Jessie"—here he exchanged a beaming smile with the girl—"an' my pension. I'm glad to think that I can live, and die, an' be buried in this village, for I am fonder on it nor tongue can tell. But you'll come down an' see us, please, an' Jessie shall show you the books Miss Nightingale giv' her, writ in wi' her own hand. I must go now an' try to get a word wi' the housekeeper." "I will come to-morrow," said I, turning towards the nut-brown maid. You must keep the books sacredly, Jessie." Her eyes sparkled, as if she knew the value of her treasures, then she took the veteran's arm and marched him off in the direction of the house, in front of which silvery waters lay sleeping. "Happy old soldier, ending his days safe from war's alarms in peaceful Claydon," and in a moment the lakes—with their background of drooping elms, the swans skimming their surface like a fleet of sail—were blotted out of sight, and again we fell to talking of Miss Nightingale's mission and its noble fulfilment. How from the time that she tended her soldiers in the rude hospitals at Scutari to this day, she is a public benefactor, sympathising with every effort for the relief of suffering, in touch with every movement for the preservation of health. As nurse, organizer, and adviser she fought with all her powers against disease and death. With voice and pen she cast out ignorance, exposed error, taught and led, till she elevated a calling and established a profession—that of the trained nurse. Her love of humanity, her devotion and enthusiasm, have their reward even here. Her name is written high upon Britain's roll of honour side by side with the greatest commanders the century has known, and it will go down to posterity as the name of one who was even greater than they. Florence Nightingale and her heroic band of helpers went out to the Crimea not to wound, but to heal—not to destroy, but to succour and to save. The light of their example has illumined the world, and by their efforts they have helped "to roll it onward toward its destined verge."

### The Princess Maud Marriage Present Fund.

Nurses sending contributions to this Fund are requested to write outside the envelope in the left-hand corner the words "Princess Maud," as this will save considerable trouble in dealing with the correspondence. Nurses are reminded that all letters on this subject should be addressed to the Manager of The Hospital, 428, Strand, London, W.C., and not to the office of the Pension Fund.

Amount previously acknowledged ... £43 17s. 6d.

Fifth List.

| | s. | d. | | s. | d. |
|---|---|---|---|---|---|
| E. Pugh ... ... ... | 1 | 0 | Nurse Dalton ... ... | 2 | 0 |
| E. M. Roberts ... ... | 1 | 0 | R. Greenwood ... ... | 1 | 0 |
| H. L. Jones ... ... | 1 | 0 | Nurse Bays ... ... | 1 | 0 |
| Nurse Waters ... ... | 1 | 0 | Policy No. 2,148 ... | 1 | 0 |
| E. Liddle ... ... ... | 10 | 0 | Policy No. 2,149 ... | 1 | 0 |
| M. Cadbury ... ... | 2 | 0 | L. Tidy ... ... ... | 1 | 0 |
| S. B. Culpin ... ... | 1 | 0 | Policy No. 4,316 ... | 1 | 0 |
| C. Nicholls... ... ... | 1 | 0 | Policy No. 602 ... ... | 1 | 0 |
| M. Puxley ... ... ... | 1 | 0 | Policy No. 2,084 ... | 1 | 0 |
| Policy No. 3,738 ... | 1 | 0 | Policy No. 2,308 ... | 1 | 0 |
| S. Nicholls ... ... ... | 1 | 0 | E. Collins ... ... ... | 1 | 0 |
| A. Faulkner ... ... | 1 | 0 | Policy No. 2,498 ... | 1 | 0 |
| A. Robinson ... ... | 1 | 0 | L. Hinton ... ... ... | 1 | 0 |
| E. B. Orchard ... ... | 1 | 0 | M. E. Hesford ... ... | 1 | 0 |
| Nurse Chapman... ... | 1 | 0 | Ada Price ... ... ... | 1 | 0 |
| Nurse Perkins ... ... | 1 | 0 | H. G. ... ... ... ... | 2 | 0 |
| Nurse Roberts ... ... | 1 | 0 | M. E. Shipley ... ... | 2 | 0 |
| Nurse Hall... ... ... | 1 | 0 | Policy No. 456 ... ... | 1 | 0 |
| Nurse Wycherley ... | 1 | 0 | B. Jones ... ... ... | 1 | 0 |
| Nurse Greenleaf ... | 1 | 0 | Nurse Tomlin ... ... | 1 | 0 |
| Nurse Halliday... ... | 1 | 0 | J. S. ... ... ... ... | 1 | 0 |
| Nurse Taylor ... ... | 1 | 0 | A. Alloway ... ... | 1 | 0 |
| Nurse Jones ... ... | 1 | 0 | Policy No. 2,043 ... | 1 | 0 |
| Nurse Pratt ... ... | 1 | 0 | G. A. Healey ... ... | 1 | 0 |
| Nurse Hopwood ... | 1 | 0 | Nurse Mayston ... ... | 1 | 0 |
| Nurse Sanders ... ... | 1 | 0 | E. C. Chawner ... ... | 1 | 0 |
| Nurse Wileman... ... | 1 | 0 | M. J. Ewart ... ... | 1 | 0 |
| Nurse Mercy ... ... | 1 | 0 | E. M. Axton ... ... | 1 | 0 |
| Nurse Marston ... ... | 1 | 0 | E. L. Chaplin ... ... | 1 | 0 |
| Nurse Hughes ... ... | 1 | 0 | H. I. I. ... ... ... | 1 | 0 |
| Nurse Pinder ... ... | 1 | 0 | A. Z. Dimond ... ... | 1 | 0 |
| Nurse Adams ... ... | 1 | 0 | M. A. Adkins ... ... | 1 | 0 |
| Nurse Hull... ... ... | 1 | 0 | Policy No. 3,907 ... | 1 | 0 |
| Nurse Knaggs ... ... | 1 | 0 | D. Buckworth ... ... | 1 | 0 |
| Nurse Thomas ... ... | 1 | 0 | Policy No. 533 ... ... | 1 | 0 |
| Nurse Thomson... ... | 1 | 0 | A. Dixon ... ... ... | 1 | 0 |
| Nurse Reding ... ... | 1 | 0 | H. E. Doughty ... ... | 1 | 0 |
| Nurse Eveson ... ... | 1 | 0 | M. Sarll ... ... ... | 1 | 0 |
| Nurse Byrom ... ... | 1 | 0 | Nurse Fry ... ... ... | 1 | 0 |
| Nurse Bowers ... ... | 1 | 0 | S. M. Bingham ... ... | 1 | 0 |
| Nurse Stuart ... ... | 1 | 0 | M. B. ... ... ... ... | 1 | 0 |
| Nurse Booth ... ... | 1 | 0 | K. Routledge ... ... | 1 | 0 |
| Nurse Mary ... ... | 1 | 0 | A. Jerse ... ... ... | 1 | 0 |
| Nurse Carter ... ... | 1 | 0 | Nurse Malvern ... ... | 1 | 0 |
| Nurse Walker ... ... | 1 | 0 | R. J. Scott ... ... | 1 | 0 |
| Nurse Baxter ... ... | 1 | 0 | Annie Lee ... ... ... | 1 | 0 |
| Nurse Cartwright ... | 1 | 0 | Policy No. 3,051 ... | 1 | 0 |
| Nurse Williams... ... | 1 | 0 | L. Dunkley ... ... | 1 | 0 |
| Nurse Shirley ... ... | 1 | 0 | Policy No. 3,563 ... | 1 | 0 |

Received per Royal National Pension Fund.

| | s. | d. | | s. | d. |
|---|---|---|---|---|---|
| A. Wilks ... ... ... ... | 1 | 0 | C. J. Wright ... ... | 1 | 0 |
| F. M. Ellison ... ... | 1 | 0 | A. Colfer ... ... ... | 2 | 6 |
| E. S. Satchell ... ... | 1 | 0 | L. E. Richardson ... | 1 | 0 |

### Glasgow Nurses' Co-operation.

The third annual meeting of the Glasgow and West of Scotland Nurses' Co-operation was well attended. It was reported that the forty-six certificated nurses on the staff had had plenty of work, and a hope was expressed that in its third year of existence the association would become entirely self-supporting. Mrs. John Elder was re-elected president at the annual meeting, and fitting acknowledgment was made as to the able management of Miss Helen Rough, the lady superintendent of the association, which has its head quarters at 18, Sardinia Terrace, Glasgow.

### Dundee Royal Lunatic Asylum.

### RECIPES FOR INVALID COOKERY.

(*Continued from page lxxxii*).

XV. Boiled Bread and Milk: Sops.—Add bread and sugar to cold milk, heat and keep boiling till the whole flows clean off the pan. Nutmeg and wine or brandy may be added.

XVI. Toasted Bacon.—Brown a slice of bread before fire, and place on cheese-toaster; then toast thin slice of bacon over the bread, and serve hot.

XVII. Egg and Brandy or Sherry.—Beat up an egg, with a teaspoonful sugar in 2 oz. cold water, to a froth, then add 2 oz. brandy, stirring till thoroughly mixed. If sherry is used, half the quantity of water only is required.

XVIII. Custard.—Beat up an egg with a teaspoonful of sugar thoroughly; then mix with a breakfast-cupful of milk. Warm in an earthenware vessel in boiling water till it becomes thick. Brandy, rum, &c., may be added as required.

XIX. Ground Rice Pudding.—Bring milk to boiling point, and gradually add rice (a teaspoonful of rice to half-pint of milk), and stir over fire until ready, or about half an hour; sweeten with sifted sugar, add a well beat up egg, and boil for another minute. May be served up hot or cold.

XX. Arrowroot Pudding.—Mix a large teaspoonful of arrowroot with a dessertspoonful of cold milk. Pour in a breakfast-cupful of boiling milk, and add a well-beaten up egg with sugar when slightly cold. May be used thus, or after it has again been boiled for 20 minutes.

XXI. Sago.—Boil gently half an ounce of sago in three-quarters of a pint of water (put on cold) for an hour and a quarter, stirring frequently and skimming when required. Sweeten with sifted sugar, and add rum, brandy, or wine as may be necessary.

XXII. Sago and Milk.—Steep the sago in either cold milk or water for two or three hours; if in water, then pour it off, and add the milk boiling, and boil till the whole becomes clear, for about half an hour. Add the egg and sugar, well beaten up gradually; and rum or brandy, &c., as required.

XXIII. Corn Flour Pudding. — Mix 2 oz. corn flour with a little cold milk into a thin paste; add egg well beaten up. Gradually heat half-pint milk, with ½ oz sugar, to boiling point; pour on flour and egg, stirring all the time; then return whole to pan, and boil for three or four minutes.

### A.—Milk Diet.

I. Breakfast (nine a.m.).—Porridge, ordinary allowance, and three-quarters of a pint or one pint sweet milk; or, gruel and bread: Oatmeal, 2 oz.; milk, half-a-pint; bread, 4 oz.; or, bread sops: Bread, 4 oz.; sugar, ¾ oz.; ¾ or 1 pint milk; or, bread and milk: Bread, 8 oz., in thin slices, and 1 pint sweet milk.

II. Dinner (two p.m.).—On Monday and Thursday—Rice pudding: Rice, 2 oz.; 1 egg; sugar, 2 oz.; milk, ½ pint. On Tuesday and Friday—Sago pudding: Sago, 1½ oz.; 1 egg: sugar, ½ oz.; milk, ½ pint. On Wednesday and Saturday—Bread pudding: Stale bread, 4 oz. grated; 1½ eggs; sugar, 3 oz.; milk, 1 pint. On Sunday—Corn flour pudding: Corn flour, 2 oz.; 1 egg; sugar, ½ oz.; milk, ½ pint. Each pudding to be served up with ½ pint sweet milk extra.

III. Supper (six p.m.).—Same choice as for breakfast.

### B.—Fish Diet.

I. Breakfast.—Same as for milk diet.

II. Dinner.—Fresh white fish, 12 oz.; whiting, sole, haddock, turbot, cod, plaice, &c., variously cooked, and when steamed or stewed to be served with the juice. Bread, 8 oz.; or bread, 4 oz, with potatoes, 8 oz.

III. Supper.—Same as for milk diet.

### C.—For Feeding with Stomach Tube.

At eleven a.m. a pint of oatmeal gruel, made with oat flour (Grant's) and milk, instead of water, and with two or three eggs added, and rum, port, or brandy, and quinine, &c., as found necessary; seven p.m., same to be repeated; or at eight a.m. milk gruel and eggs as above; two p.m., custard, three eggs, and pounded biscuits in place of oat flour, with strong beef tea, or any of the various preparations of extract of meat, fluid meat, Bovril, &c., in all one pint to a pint and a half, with brandy, wine, or rum, and quinine, &c.; eight p.m., milk gruel and eggs as above.

### Help the Nurses to Help the Sick.

"Do not crouch to-day and worship
The Old Past, whose life is fled;
Hush your voice to tender reverence;
Crowned he lies, but cold and dead.
For the Present reigns our monarch,
With an added weight of hours;
Honour her, for she is mighty;
Honour her, for she is ours!"
—*Adelaide Procter.*

Year after year we appeal to our readers for Christmas offerings for the sick, the sorrowful, and the destitute, and we are proud to say that we do not appeal in vain. From the many valuable societies to which reference is constantly made in our columns, it is not difficult for our readers to make their own selection of destinations for their alms. We do not assume any right to dictate, but only suggest some of the directions in which seasonable aid is needed. Will our readers "help the nurses to help the sick" by interesting everyone they know in some particular institution? In that case the gifts this year will assuredly exceed those of any previous Christmas.

**The Royal National Pension Fund for Nurses,** 28, Finsbury Pavement, London, E.C.—The year 1895 has been the most successful and satisfactory since the establishment of the fund, both as to proposals and policies issued, and from the increase in revenue. Over 250 more proposals were received than during 1894, and the policies issued are already 200 more than in 1894. In sick pay some £850 have been distributed. The year has further been remarkable on account of the reception of the third and fourth thousand nurses at Marlborough Houss by H.R.H. the Princess of Wales, the president of the fund; and by the magnificent addition to the Donation Bonus Fund, amounting to £23,500, given by the merchant princes of London. The fund sustained a great and sad loss by the death of Dr. J. S. Bristowe, one of the members of the council, and late senior physician of St. Thomas's Hospital. Dr. Bristowe always displayed the keenest interest in the progress of the fund, and was ever ready to do his utmost to promote the welfare of the nurses by showing them how greatly to their advantage it was to join from the first. Dr. Herbert P. Hawkins, one of the assistant physicians of Thomas's Hospital, has been chosen by the council to fill the vacancy caused by the lamented death of Dr. Bristowe. It is a pleasure to note the addition to the council of the names of Mr. Walter S. M. Burns, Mr. Eric Hambro, and the Hon. Walter Rothschild, thus assuring a continuance of the great interest taken by their respective fathers in the welfare of the Pension Fund. Miss Mary L. E. Dunn (the superintendent of the Q.V.J.I. in Dublin) and Miss Nott-Bower (matron of Guy's Hospital) were elected as annuitants' and policy-holders' representatives at the election last March.

**The Junius S. Morgan Benevolent Fund,** which is an auxiliary to the above fund for nurses, was founded through generous contributions from nurses themselves, and raised to handsome proportions by the munificence of the Morgan family and many other friends to nurses. It has already dealt with numerous cases of nurses who, without this aid, would have been destitute. The business of the Royal National Pension Fund is admirably managed by men of knowledge and experience; the work of the Benevolent Branch is done by volunteers, under the supervision of an influential committee, of which Lady Rothschild is President, which devotes time and care to the investigation of claims and the relief of urgent cases. About 28 nurses are now life pensioners, and no nurse is permitted to leave the Pension Fund until every effort has been made to afford her assistance to tide over her difficulties. Fresh subscriptions are now required to extend help to those outside the Fund. Hon. Secretary, Miss Rosalind Pritchard.

**The Invalid Children's Aid Association.**—This ideal charity has its offices at 18, Buckingham Street, Strand. The object of the association is to help invalid and crippled children in their own homes. A system of regular visiting is organised, and the ladies who volunteer for this work find out what is most needed by the little people, and endeavour to supply it. Spinal carriages, perambulators, surgical instruments, and bed linen are lent, and clothes, toys, picture books, &c., are given. Tickets for seaside and country convalescent homes are distributed—in fact, much useful work is unobtrusively done by this association. Funds are greatly needed by the committee to enable them to carry on their work. Subscriptions are gladly received by the Hon. Secretary, Mr. Allen Graham.

**Workhouse Infirmary Nursing Association.** Offices, 6, Adam Street, Strand.—Much progress is being made in introducing an improved system of nursing into workhouse infirmaries, but much yet remains to be done. The association has trained 250 probationers, and 130 nurses are now at work. In many cases the superintendent and staff are supplied to country union infirmaries. Increased financial support is urgently pleaded for, to extend the work of training probationers, to which purpose the greater part of the funds are applied. The objects of the association are: To raise the standard of public opinion on the whole question of workhouse nursing; to secure the appointment of trained ladies as matrons in all separate infirmaries; and to train and supply nurses to workhouse infirmaries in London and the provinces. H.R.H. Princess Mary Adelaide of Teck is President of the association, and subscriptions will be gladly received by the Hon. Secretary, Miss Wilson, 13, Barkston Mansions, S.W.; or they can be paid in to the account of the W.I.N.A., at Lloyds Bank, 222, Strand, London.

**The Ladies' Sanitary Society.**—This society, deserving of every encouragement, was founded in 1859, and at its first meeting Charles Kingsley called it "one of the noblest, most right-minded, straightforward, and practical conceptions that he had come across for some years." Particulars of the work of the society can be obtained from Miss Rose Adams, the Secretary, 22, Berners Street, London.

**St. Marylebone Female Protection Society,** 157, 159, Marylebone Road, N.W.—This institution is one of the oldest charities in London, and is engaged in rescuing young women who have been led astray. To send such women to the workhouse means further degradation, and therefore this society steps in. Highly gratifying results have attended its operations, as may be seen by the annual reports. Contributions are much needed, and should be sent to Mr. George Scudamore, Secretary.

**Irish Distressed Ladies' Fund,** 17, North Audley Street, W. Patron, the Queen.—Relief is given independently of any question of politics or religion; employment is found when possible, for those able to work; pecuniary help is given to the aged and infirm; and the education of children is paid for. Secretary, General W. M. Lees.

**Princess Louise Home, National Society for the Protection of Young Girls,** Norbiton, Kingston Hill, Surrey.—This society is the parent of all others of a similar character in the United Kingdom, having been founded in 1835. It has been the means of rescuing close on 1,700 young girls from bad surroundings, and has placed about 1,200 in service. It has 100 under its care at the present time, and is certified for 150 by the Local

Government Board. The schools are under the Education Department, and the maximum grants are invariably earned in each subject of examination. A laundry is attached to the home, by means of which a fairly good amount is earned towards the support of the institution. A system of marks prevails here, carrying with them pocket-money and small salaries, which are banked for the girls and made over to them when they start life on their own account. Funds are very urgently needed to clear the debt still owing on account of building and furnishing the laundry three years ago, and also to enable the committee to take in the full number of girls the home can accommodate.

**Metropolitan Nursing Association,** Bloomsbury Square, W.C.—Founded in 1875 as a Training School and Home for District Nurses who have previously, gone through a full course of hospital training, and who nurse the sick poor in their own homes within a radius of a mile and a half from Bloomsbury Square. This Association is now the central training home for the Queen's Nurses. Superintendent, Miss Gray.

**Queen Victoria's Jubilee Institute for Nurses.** Offices at St. Katharine's Hospital, Gloucester Gate, Regent's Park, N.W.—The institute trains nurses in district work and supplies nurses trained in district as well as hospital nursing for the sick poor in their own homes. Applications for information should be addressed to Miss Peter, the Inspector. Scotch, Irish, and Welsh branches also exist, and numerous associations have affiliated themselves with the Institute.

**"The Hospital" Convalescent Fund.**—Since the first establishment of a nurse's free bed many tired and delicate workers have enjoyed a much-needed change of air such as they could not possibly have secured for themselves without help. Experience has proved that it is better to let the nurses have a choice of locality rather than to send them to one settled place, and therefore the new title of The Hospital Convalescent Fund has replaced the "Nurses' Bed." The object is, however, the same, namely, the provision of rest for weary workers, amidst suitable surroundings, without any of that anxiety about ways and means which retards convalescence. The fund is a small one, and contributions which would increase its field of usefulness are invited by the Hon. Secretaries, care of the Editor of The Hospital.

**Our Needlework Competition** results each year in a number of useful garments being forwarded to many hospitals. We receive and distribute parcels of excellent warm clothing made by readers, who would feel amply rewarded if they saw the pleasure which their kindly offerings give. The demand for suitable garments for poor patients discharged from hospital is very great. The obvious necessity for persons recently inmates of sick wards being properly clothed needs no comment, and liberal support should certainly be given towards this object.

### Appointments.

Metropolitan Idiot Asylum, Darenth.—Miss E. E. Penney, of the Infirmary, Dulwich, has been made Matron of this asylum. We wish her every success.

Manchester Royal Eye Hospital.—Miss Caroline H. Glover has been appointed Matron of this hospital. She was trained at St. Thomas's Hospital, and was afterwards sister of the ophthalmic ward. Miss Glover then worked for a year at the Hospital for Sick Children, Great Ormond Street, and has held the post of matron at the School Sanatorium, Shrewsbury, since January last. We congratulate Miss Glover on her appointment, and wish her every success in her new work. She holds excellent testimonials.

### Christmas and New Year Gifts.

Now that Christmas is almost upon us, our thoughts turn towards the little presents appropriate to the season. Few nurses have many leisure hours at their disposal in which to seek suggestions from the shop windows in all their bright gala attire. Indeed, so many alluring shows distract rather than help us to a choice, and it is best to know what we want before we start out. As our Christmas Supplement appears early this year, we are able to take the opportunity of offering a little help to our readers and especially to those who live far away from the regions of shops, and have to make their purchases through the post.

### Presents for Invalids.

For invalids there are many things which suggest themselves to our mind as appropriate and useful. Handkerchief sachets made of fine white linen, or some kindred material, with delicately-traced sprays and ornamental letters worked across them, are being displayed at Harris's Art Linen Show Rooms, 25, Old Bond Street. One of these would form a dainty and useful gift, especially if stored with some of Robinson and Cleaver's Irish linen handkerchiefs. Those entitled "Sweet Lavender" have become extremely popular, and have been purchased by Royalty. Messrs. Harris design these in different sizes and colours; a lovely one can be bought for 5s. 6d. Then, again, that staple commodity, a bottle of Eau de Cologne, is always appreciated in the sick room. The best Eau de Cologne "4711" is to be had at 62, New Bond Street. This scent has a special quality of its own, and our readers will agree we have not been too cordial in its praise. Mr. Reuter, the agent of the celebrated manufacturer at Cologne, asks 2s. 6d. a bottle for this, but cases of half-a-dozen may be had at a reduced rate, and the carriage is free. Another delicious scent supplied by this firm, "Rhine Violets," has attained almost equal popularity, and is also suited from the delicacy of its perfume to the sick-room. A hot water bottle is another serviceable and welcome gift; indiarubber ones are the most convenient, but they should be obtained of the best quality. Messrs. Anderson and Anderson, of 37, Queen Victoria Street, E.C., we can safely recommend as supplying most reliable ones, and one we have ourselves had in use for four years is still flawless and strong. Another comfort for an invalid is a little bed-jacket to keep the arms and shoulders warm. A knitted shoulder-cape trimmed with chenille is very suitable for this purpose, as it is easily slipped on and off, and is both elastic and light. The Knitted Corset Company (of Nottingham) supply these handy little capes. Talking of warm, cosy bed-room apparel, Fleming, Reid, and Co. (84, Oxford Street) should be consulted through the medium of the catalogue which they supply upon application, and which will be found full of alluring suggestions. Fleming, Reid, and Co., of Greenock, are deservedly celebrated, as their productions are of unusual excellence and durability.

In the way of sick-room literature we have something quite new to introduce to our readers just brought out by the Religious Tract Society. This is the novel experiment of printing entertaining short stories, hymns, &c., on linen strips for the special convenience of those who are unable to hold up heavy books or turn over their pages. So far the choice of linen leaflets is not a large one, but no doubt the society will greatly extend their store as the series becomes better known. They will be pleased to send our readers a list of what are now ready.

The Oxford Clarendon Press has recently brought out some lovely miniature editions, printed on India paper in thumb size, of "The Christian Year" and "The Imitation of Christ," from 1s. to 1s. 6d., according to the binding. All booksellers sell these.

Presents for Friends.

A present that is always appropriate is a calendar. Especially beautiful designs are produced this year by Marcus Ward. The same firm have diaries of every description, both large and small, which are appropriate for New Year gifts.

Stationery is another very acceptable gift at the present season of much letter writing. We were much struck the other day with some correspondence letter cards manufactured by Messrs. Beeching, in the Strand. These are of ingenious and unique device for saving time and trouble.

As a present to a nurse a "wallet" is always useful, for the wear and tear of ward life soon takes off the freshness of the best, and then a new one is very welcome. A great variety of these are to be seen at Messrs. Allen and Hanbury's, Garroulds', and at Bailey's, 38, Oxford Street. They are to be obtained in tortoiseshell or morocco, and in some cases they are so constructed that the instruments are kept dust-proof, with detachable straps, making them into a compact little pocket-case. "The Nurse's Dictionary," by Honnor Morten (published by the Scientific Press, 428, Strand), is a fitting accompaniment to the wallet. The little book serves the useful purpose of explaining medical terms, and is designed to be used by the bedside as a temporary reference book until there is time to look up fuller works on the different subjects. We should like to call attention to a nurse's watch, which we have tested ourselves and found most satisfactory. It is manufactured in Switzerland, with all the recent improvements for winding without a key and altering the watch without opening it. It is jewelled in eight holes, and has the second-hand. This watch is most admirably adapted for nurses' use, and is to be had of J. N. Masters, Rye, Sussex, for the sum of £2 2s. Besides these, Messrs. Harris have a great variety of calendars, notebooks, tea cosies, nurses' aprons, &c., embroidered in their lovely art linens, which also have the charm of the latest novelties.

Nurses are often consulted on the subject of presents by their patients. If a useful gift is wanted for a medical friend, a very acceptable one will be found in a medical diary. One we have seen published by Messrs. John Wright and Co., Bristol, has the special advantage of showing a whole month's entries in an opening, and also has a "perpetual" visiting list (a useful innovation), and is provided with many useful tables and information. The whole closes in a neat wallet, and can be purchased for the price of 5s. 6d.

Presents for Children.

In many hospitals and other institutions where the amusement of the young is provided for at Christmas the Christmas tree or a bran pie is a common feature. The collection of the many and various toys necessary is a great tax on the purchaser, who is not infrequently an over-busy matron or nurse. Most of the large drapery establishments have large bazaars at this time of the year, which greatly reduce the difficulty of making a choice, Messrs. Whiteley and Messrs. William Owen, in Westbourne Grove, and Messrs. D. H. Evans and Co., in Oxford Street, are especially to be mentioned for their brilliant Christmas bazaars, where toys to suit all fancies are to be found at absurdly low prices. At Messrs. Evans' in Oxford Street the display is unusually varied and tempting, and would-be purchasers should go in good time, or they may have to wait some time for the delivery of their purchases. At Peter Robinson's, in Oxford Street, a special feature is a collection of 140 different games.

Presents for the Poor.

Not infrequently the district nurse is asked to act as almoner by well-to-do friends, or to assist with advice, for she has full means of knowing the wants of the poor who are her patients. She would often like to ask for much-needed blankets, but these form rather expensive presents, and restrict the use of a given sum to too few recipients. This difficulty is now removed by the manufacture of really excellent cotton blankets, so like woollen ones both in appearance and substance that the difference is hardly perceptible. They are found very nearly as warm, and very little heavier, whilst the cost is about half that of their rivals. We are told they wash well. We imagine they can be obtained at most drapers', but if any reader has a difficulty in securing them we shall be glad to send an address.

Christmas Cards.

Christmas cards have not yet had their day, and there is still so great a demand that most publishers present new designs each year. It is noticeable that the general taste in Christmas cards is veering towards a preference for the quieter-toned varieties. Messrs. Marcus Ward have a delightful selection which will find favour amongst the more sober-minded purchasers. "The Angels' Song," one of a kindred series, is composed of beautiful verses, printed on the best paper, illustrated by appropriate etchings, and enclosed in a cover of artistic design. Such Christmas or New Year's mementos are really worth keeping, and, therefore, worthy of sending. There are many others too numerous to mention, but Messrs. Marcus Ward's catalogue should be consulted, as it will be found very useful to country purchasers; for if those we have selected for description do not suit their fancy, they will find something to please all tastes within its pages, where many designs will be found illustrated. "Marcus Ward, London," will be sufficient address, and then any cards or other articles selected can be ordered from a local shop, if desired. At Messrs. Beeching's, in the Strand, there are also some pretty Christmas cards to be obtained, with artistic designs of London; and other correspondence cards with scenes from London life are also original and pretty.

### Royal British Nurses' Association.

The eighth annual conversazione of the Royal British Nurses was held at the Royal Institute of Painters in Oil Colours on Monday evening last. The galleries were thronged by guests, who were received at the entrance by Miss Thorold, matron of the Middlesex Hospital, Sir James Crichton Browne, Mr. Langton, Mrs. Dacre Craven, Miss Ravenhill, and Mr. Fardon. The scene was a pretty one, with the moving mass of kaleidoscopic colours, brilliant evening dresses and picturesque uniforms, the white caps and aprons of the nurses giving just the relief required, and the pictured walls providing a charming background. The lighting by gas, and the apparent absence of ventilation, combined with the fact that the numbers of people present were far too many for the space, must have militated against the enjoyment of a good many—the heat being overpowering. A very pleasant musical and dramatic programme was provided, in which Mr. Charles Collette, Mr. David Bispham, Mr. H. Wharton Wells, Mrs. Bancroft, Miss Olliffe, and Miss Rollo took part. The Lady Mandoline Guitar Players, conducted by Signor Zeriga, also played during the evening. An electrophone too, was laid on to various theatres and music halls, &c., which, to judge by the crowd awaiting admission, proved a popular source of amusement. The refreshments were, as usual, excellent.

Amongst the guests were the Lord Chancellor and Lady Halsbury, Lady Dorothy Nevill, Sir Richard Quain, Sir Dyce and Lady Duckworth, Mr. Pickering Pick, Mr. Mark Hovell, Miss Isla Stewart, Miss Smedley, Miss Campbell, Mrs. Coster, Dr. and Mrs. Bedford Fenwick, Mr. and Mrs. Melhardo, and Miss Peter.

### Christmas Books.

Mr. H. C. Beeching has selected a rare garland of Christmas verse in his recent volume, which, adorned by the graceful designs of Mr. Walter Crane, will afford the lover of poetry many hours of contented browsing among its pages. The range is a wide one, excluding only what Mr. Beeching calls "the poetry of Entire," which draws its inspiration from the beer barrels; but we are glad to find wanting also some too conspicuous favourites of the choir-boy. Nothing can be more interesting than to trace the development in English poetry through five hundred years of the special inspiration supplied by the Christmas season. After a few fervid Latin hymns, dating back to early times (the date assigned to the Adeste Fideles is an obvious misprint), are some of the earliest English carols. "This Endris Night," dating from the reign of Henry VI., may have been a popular cradle song. It begins—

"This endris night
I saw a sight,
A star as bright as day;
And ever among,
A maiden sung
Lullay, by-by, lullay.

This lovely lady sat and sang, and to her childë said,—
'My son, my brother, my father dear, why liest thou thus in hay?
My sweetë bird, thus it is betid,
Though thou be king veray,
But, nevertheless, I will not cease
To sing lyly, lullay.'"

Some of the most charming of these carols are cradle songs, of which the rhythm and form peculiarly suit the subject; the "Rocking Hymn" of George Wither is a good example, and has a pretty and tender illustration. To Milton's "Ode on the Nativity," the most telling of all these fine designs is appended, the angels slanting across the sky in long luminous lines like moonbeams, directly over the heads of the shepherds, who are straining their sight to behold the vision. From the mystic rapture of Robert Southwell and the involved introspection of Herbert and Donne, the verse passes to the varied melody of modern times, least pleasing perhaps in the archaic splendour of Swinburne, and blossoming into something like the gracious simplicity of the earlier carols in the hands of Christina Rossetti. The volume is most attractively bound and printed.

The passion for dialect has invaded the book world of late to such an extent that the vulgar preference for books dealing with dukes and duchesses rather than poor people is beginning to spread, since the reader is sure, in "high society" at all events, of escaping from a new world of vowels and abbreviations. "By Thrasna River" is, alas! written much in dialect, but though the usual struggle to spell every word a little bit differently to the common use is maintained, it must be conceded that Irish is a tongue more easily grasped by the stranger than the northern idiom, and few will object to a copious sprinkling of "Och, whist," and "Arrah!" where the meaning of the text can be seen at a glance. The story relates the adventures of a young Irish student who failed in his final examination and took refuge in the study of farming, love-making, and fighting at Emo. There is much country humour and pathos in the story, and the character of Harry Thompson, the self-taught lover of books, and the tragedy of his love and ambition are well sketched.

The Nautilus Series is a handy set of novels, attractively presented at a very moderate price. "A Comedy of Honour" relates the complicated emotions of two engaged couples on discovering they were wrongly matched and planning a rearrangement of hearts. The sentiment is cleverly kept on a low note, so that the play of what Meredith calls the "nice shades and fine feelings" never becomes fatiguing, and the *dénouement* is brought about with some considerable skill and humour.

Everyone will welcome the new hand-book to crime in the shape of the second series of "The Chronicles of Martin Hewitt, Investigator," who has now become a serious rival to the immortal Sherlock Holmes. Here may be studied a new and ingenious process for hiding stolen diamonds, for stealing an inconvenient will, for constructing a "hand of glory," for secreting bullion in a cabbage garden, for hocussing a bank clerk, and, finally, for making explosive cottage loaves. Of course, in every case the criminals are mere babes in the hands of Mr. Hewitt, whose knowledge of London life, like that of Mr. Pickwick, is extensive and peculiar, but whose clues are sometimes a little too dependent on good luck to give the other si e a fair chance. Not a very high form of fiction perhaps, but unrivalled in its power of lending relaxation to a tired brain.

The *Quiver* presents its usual handsome appearance, and all who have any experience in lending libraries know how highly its varied contents and profuse illustrations are valued. This year forms no exception to its ordinary high standard of merit.

"A Book of Christmas Verse." Selected by H. C. Beeching, with ten designs by Walter Crane. (Methuen and Co.)

"By Thrasna River." By Shan F. Bullock. Illustrated by St. Clair Simmons. (Ward, Lock, and Bowden.)

"A Comedy of Honour." By Nora Vynne. (Ward, Lock, and Bowden. Nautilus Series.)

"Chronicles of Martin Hewitt." By Arthur Morrison. (Ward, Lock, and Bowden.)

The *Quiver*. (Cassell and Co. 1895.)

### Books for the Young.

An unusually pretty and interesting collection of gift books is published this year by the National Society, and a visit to their depository in Broad Sanctuary, Westminster, should enable the most fastidious donor of prizes to satisfy every variety of taste at wonderfully small cost. Miss Yonge, in spite of the crowds of modern competitors, is still a name to conjure with, and her new story, "The Carbonels," with illustrations by W. S. Stacey, of which we append a specimen, is sure of a wide circle of readers. It is well to be reminded within how short a period the reign of education and social improvement has been established in rural England, and this picture of the villagers' life in 1822—without schools, or clergyman, or doctor, and under the blasting influence of the old poor law, with Jack Swing in the background—is a wholesome object lesson for admirers of the good old times. No one knows better than the authoress the difficulties and disappointments of village work, religious and social; striving, forbearing, and hoping still, as she has done for over half a century in her own village home, "like a tree planted by the waterside that will bring forth his fruit in due season." But in the teeth of all discouragement the keynote of her later as of her early works is still undaunted faith in the ultimate triumph of Christian endeavour, and the glance backwards from the standpoint of 1880, with which this volume closes, enforces the motto she has chosen from her old friend and rector, John Keble :—

"God hath sown and He will reap ;
Growth is slow when roots are deep ;
He will aid the work begun
For the love of His dear Son."

A charming tale, by Mary H. Debenham, relating the dealings of two very juvenile maiden aunts with their little nephew, is also drawn from the beginning of this century, but here the picture is *couleur de rose* indeed. Everyone is perhaps a little bit too good for this work-a-day world, but that is a fault which young readers never object to, provided there is, as in the present case, plenty of fun and adventure, with a spice of pathos and romance to make the mixture slab and good.

"Dorothy's Stepmother" is rather an ordinary tale on a well-worn theme, but it is amusing to note in the modern story-book how completely the stepmother has been whitewashed. In fact, the injustice is now all the other way, and we find ourselves wondering how these elegant young women can be induced to assume the direction of the regulation stern and peculiar story-book father and his ill-behaved offspring.

A good historical story of the Thirty Years' War, by M. Bramston, will be welcome in many schoolrooms where this difficult period has been under discussion. The authoress has wrestled successfully with a complex subject, and has succeeded in presenting a sufficiently lucid narrative likely to capture the interest of her readers, and with enough preparatory matter to enable them to follow the event without much previous knowledge of the period.

"The Artist of Crooked Alley" is a conventional story of a picturesque little "screever" or street artist, who rose by aid of good conduct and rheumatic fever to the somewhat depressing altitude of drawing master in a provincial town. It is prettily told, but alas ! little London boys are not like this.

"A Puff of Wind" deals with the stirring days of smugglers and highwaymen. The interest is well sustained, although the plot is rather thin and the characters shadows.

"Stories for Ten-Year-Olds," by F. W. Saunders, has almost every fault that a child's book can be guilty of. The stories are so slight as hardly to deserve the name, the style is stilted and cynical, and the perpetual jocular allusions, social, moral, and religious, while totally incomprehensible even to the modern child, are in the worst possible taste. It is hard to say which of these pages it would be most painful to find a ten-year-old studying—the stable talk and betting slang of Ladas, the irreverent travesty of Sir Ga'ahad, and of Adam and Eve, or the exquisite humour of the little girl who tried to bury her baby brother alive when she learnt he would be the heir. But doubtless the ten-year-olds may be safely trusted to reject with all the happy peremptoriness of their age a book which is so strangely unsuited to the child-like mind.

The only flaw in "Master Jimmy's Fables" is that they provoke a tendency in every one who gets hold of them to read them aloud in a high tone at railroad speed, to the irritation of peaceable bystanders and the confounding of rational conversation. There is certainly a great fascination about them and in the very unconventional morals which Master Jimmy deduces from his own versions of the stories he has been accustomed to hear. In all probability the nursery will soon get to gabble them all off by heart, and the clever illustrations by Walter Warren are sure to be very popular, and become only too well acquainted with the new Christmas paint-boxes.

"For Glory and Renown," by D. H. Parry, is a capital story of fighting and adventure in the eighteenth century. The hero, starting well with a boar hunt, wolves, smugglers, and a fair lady in a cave, with a subterranean passage to a ruined tower, goes on to the even more stirring incidents of the taking of Quebec, the capture of an English man-of-war by French privateers, and so much fighting and bloodshed that the marriage and the earldom with which the end is reached comes almost as an anti-climax. It can be well recommended for the large class of school-boys who decline to read at all except on strong provocation.

The fourth series of "The Zoo," by the Rev. Theodore Wood, makes a very attractive volume. It is mainly taken up with water fowl and the reptile house, but there are also charming illustrations from the parrot-house, the bower birds and the pheasants. Some humorous sketches of the adjutant will be of special interest to the fortunate possessors of "The Second Jungle Book."

From the S. P. C. K. comes also the annual volume of the "Child's Pictorial," full of gay pictures and stories for quite the tinies. "The Dawn of Day," published by the same house, is a familiar and welcome presence in many parishes, and makes a handsome volume in its deep scarlet binding.

"Little Folks," in the gayest of gay bindings, is as attractive as ever, and caters with its usual success for a wide circle of readers, ranging from six to sixteen. The puzzle pages and competitions are specially ingenious, and many of the illustrations show a high order of merit.

The Christmas number of "Bubbles," Dr. Barnardo's coloured magazine for children, contains, amongst other seasonable stories, one by H. F. Gethen, entitled "How Michael got admitted to Hospital."

"The Carbonels." By Charlotte M. Yonge. With five full-page illustrations by W. S. Stacey. Price 3s. 6d.

"Two Maiden Aunts." By Mary H. Debenham. Price 2s.

"Dorothy's Stepmother." By Penelope Leslie. Price 1s.

"The Story of a Cat and a Cake." By M. Bramston. Price 2s. 6d.

"The Artist of Crooked Alley." By Andrey Curtis. Price 1s. 6d.

"The Puff of Wind." By F. C. Badrick, with illustrations by J. Staniland. Price 1s. 6d.

(National Society, Westminster.)

"Stories for Ten-Year-Olds." By Frances Wilce Saunders. (Swan Sonnenschein and Co.)
"Master Jimmy's Fables." Collected by St. John Browne. Illustrated by Walter Warren. (The Leadenhall Press, Limited, 50, Leadenhall Street, E.C.)
"For Glory and Renown." By D. H. Parry. (Cassell and Co., Limited).
"The Zoo," Fourth Series. By the Rev. Theodore Wood, F.E.S.
"The Child's Pictorial." A monthly coloured magazine. 1895.
"The Dawn of Day." For Sunday-school and parish use. 1895. (Society for the Promotion of Christian Knowledge.)
"Little Folks." New and enlarged series. 1895. Price 3s. 6d., or cloth gilt, 5s. (Cassell and Co., Limited.)

### Notes and Queries.

### Queries.

The following is a specimen of the curious questions propounded to an Editor of a medical journal : I should esteem it as a favour if you would kindly inform me to what hospital, society of surgeons or other institution one should apply in order to mortgage one's body for a fixed sum of money?—*A. W. C. H.*

(53) *Pensions.*—Please give me the address of the Nurses' Pension Fund Office.—*A. L.*

(54) *Registered Nurses.*—Where is the office of the Registered Nurses' Society, and how much does it cost to become a member ?—*H.*

(55) *L.O.S.*—Many nurses are anxiously interested to know whether Dr. Davison's as ertion to the Spalding Guardians is accurate. He was reported to say there that certificates granted by the L.O.S. "were of no value at all ; in fact, they were illegal." Nurses are therefore anxious to hear from the Editor of The Hospital the truth of this statement.—*A Certificated Midwife.*

(56) *Nurses' Co-operation.*—Can you tell me the address of the office where, I am told, trained nurses only are employed for private nursing ? I heard they took all their own earnings, so I suppose they pay some entrance fee. Can you kindly tell me this ?—*Country-trained Nurse.*

### Answers.

(53) *Pensions (A. L.)*—The offices of the Royal National Pension Fund for Nurses are at 28, Finsbury Pavement. Secretary, Mr. Louis Dick.

(54) *Registered Nurses (H.)*—No. Regent Street. You had better apply there yourself for particulars. Nurses pay an entrance fee, and have to provide uniform, &c.

(55) *L. O. S. (A Certificated Midwife).*—You and your friends will find but little maternity work is now given to nurses who do not hold this certificate or an equivalent. You need not trouble yourself about statements such as the one you quote, as long as so many boards of guardians and other employers think differently. Because a certificate does not constitute a legal qualification, it is not necessarily an illegal or worthless one. It is untrue to say that the certificate granted by the L.O.S. is "of no value at all," and equally so to say it is "illegal." Any nurse applying for any maternity work will soon find how her way is smoothed by the possession of it or of an equivalent, and it certainly is not "illegal," although it conveys no legal rights.

(56) *Nurses' Co-operation (Country-trained Nurse).*—Apply to Lady Superintendent, 8, New Cavendish Street. There is no entrance fee.

### Everybody's Opinion.

[Correspondence on all subjects is invited, but we cannot in any way be responsible for the opinions expressed by our correspondents. No communications can be entertained if the name and address of the correspondent is not given, or unless one side of the paper only be written on.]

### CHRISTMAS DECORATIONS IN HOSPITALS.

"An Old Nurse" writes: With much regret do I find that one of your correspondents is discouraging decorations. I agree with the reasonable remarks made recently in The Hospital, for I do not think nurses can afford to spend much time or money on such things. But don't let us be deprived of the pleasure of giving up a little of both to please the sick and helpless. Christmas would lose half its pleasure for me if I couldn't have a bit of holly about! Perhaps Mr. Holland has never helped to take down the decorations, as many kindly students, aye, and even the residents, have done. Had he assisted at this rather dusty job, he would know that even abused evergreens are reduced by three or four days' residence in hospital to a shrivelled and brittle condition. I have never seen decayed vegetable matter on the walls of any institution in which I have had the honour of spending Christmas.

"A House-Surgeon" writes: As one who has held surgical resident appointments at East-end hospitals for over four years, I should be much obliged if you would allow me to raise a word of protest against the remarks of the Hon. Sydney Holland in his letter in the issue of The Hospital for December 7th, 1895, with regard to Christmas decorations. At the hospital at which I have the honour to be house surgeon, the chairman of the committee would not, I am sure as a layman, express any opinion as to whether Christmas decorations were sanitary or not; and instead of there being "not the slightest doubt" (as the Hon. Sydney Holland says) that they are necessarily insanitary, I am quite certain that there is every doubt about it. Of course, if the Hon. Sydney Holland's only idea of artistic decoration lies in the direction of covering walls with decaying vegetables (*sic*), the less of any decorations carried out according to his ideas the better. But he carefully ignores the much more artistic and sanitary methods of decoration by means of drapery, &c., coming more and more into vogue at Christmas, in which the patients are as much interested and, if possible, even more pleased than they used to be with the "decaying vegetable" method. Let me also assure the Hon. Sydney Holland that no nurse is ever forced to help in the decorations, if she wishes to be doing anything else. His objection with regard to the expense comes very artlessly from the chairman of a hospital which is just now appealing for £3,000 for the erection of isolation accommodation for a service of only sixty or eighty beds.

### A CAP AND BELLS.

"A Nurse and Englishwoman" writes: I have been amused at the correspondence which has gone on for many weeks in The Hospital anent the "Abuse of Uniform," and my own impression is that nurses have only themselves to thank for such "abuse" being possible. If they would keep their working dress for their work, as other people do, they would be no more troubled with imitators than those who do not rush into print crying for "Protection," demanding that the private and variable thing called "nursing uniform" should be put upon the same footing as the Queen's. Were such an enactment possible, any individual who started a nursing home or a district nurse would have the power of making a sumptuary law that nobody else was to wear such garments as they chose to style their uniform, and no woman could venture to buy a dust cloak until she ascertained that its make had not been monopolised by some nursing institution. What can outsiders think of the common-sense of women who want the Princess of Wales to do for them what she certainly could not do for herself; and who wear uniform because otherwise they would have to spend all their salaries in dainty shoes, &c., and spend half-an-hour in changing a dress? If we nurses are so feckless and helpless that we must spend all our wages in dressing as decently as thousands of women do who have not half as much to spare, and require half-an-hour for doing what others can do in ten minutes, I think that a "cap and bells" would be at once our most fitting badge, and one which imitators would let severely alone.

### THE JOY OF CHRISTMAS.

"A Ward Sister" writes: I trust that the editor of The Hospital does not endorse the sentiments, or rather lack of sentiment, of the gentleman who wrote in last week's issue? I hope the day will never come when nurses, as a body, grudge putting a little beauty into the unlovely lives of the poor. Many a truly happy Christmas have I spent with them, not as a prosperous visitor looking at completed "effects," but working hard with and for the patients. Is it not more than sufficient reward for a little extra trouble when a sick man or woman bids one "God-speed" in one's labours, and thanks one (with tear-dimmed eyes) for "all the trouble as you've took to make things pleasant at Christmas."

### NURSES' UNIFORM.

"Spatula" writes: May I suggest that every trained nurse wears a badge, Government marked, with her own name engraved on it? To procure this she must produce her hospital certificate. The suggestion that nurses should wear a certain uniform would be no preventative of imitation, as anyone might copy it; besides, institutions would not allow those who were policyholders to dress differently to others.

[We do not see how either uniform or badge could prove the wearer to be a trained nurse, nor do we see what is to be gained by thus labelling those who follow a calling. In other branches of women's work it is not thought necessary to hall-mark experts. Why should nurses be either legally or fancifully "stamped"?—Ed. *T. H.*]

### A MISUNDERSTANDING.

"T. M. P." writes: In the Nursing Supplement of the 16th ult. appeared an advertisement headed "New City Hospital, Little Bromwich." My daughter, a trained nurse, who had just completed her course of midwifery at the Maternity Hospital, Glasgow, answered that advertisement, and somewhat promptly received a reply engaging her and asking her to enter upon her duties forthwith. She, therefore, went to Birmingham on the 28th ult., and I have a letter from her telling me that on her arrival she discovered that the institution was a fever hospital, and on complaining to the matron and the medical superintendent, was told by the former that "city hospital" was always understood to mean fever hospital, and by the latter that he thought he had mentioned in his letter that it was a fever hospital. I can testify that there was nothing whatever either in them or the heading of the paper to show that the hospital was one for the treatment of infectious diseases. Had either my daughter or I known the nature of the institution beforehand she would have declined the engagement, and I should have most certainly sanctioned her doing so. She feels that she has been the victim of a trick, and I have no doubt that if she chose to do so she might leave at once and recover her travelling expenses, amounting to about £2 10s. I shall be obliged if you will be good enough to insert this in your next issue as a warning to other nurses who may be misled by similar advertisements.

[It seems to us that our correspondent's daughter must have accepted this engagement without troubling herself to make any enquiries as to the institution she was entering. She had better make use of the opportunity which she has unintentionally secured and gain experience in fever nursing which she cannot fail to find valuable hereafter. The prefix "City," "Borough," or "Municipal" is usually understood to mean that a hospital is for infectious diseases. With regard to what our correspondent considers misleading advertisements, we advise him in future to call his daughter's attention to "Burdett's Hospital Annual," in which all existing institutions appear under proper headings.—Ed. *T. H.*]

